# Clinical outcomes of an ultrathin-strut sirolimus-eluting stent in all-comers population: Thailand Orsiro registry

**DOI:** 10.1186/s12872-021-02310-0

**Published:** 2021-10-16

**Authors:** Pannipa Suwannasom, Siriporn Athiksakul, Tasalak Thonghong, Vorarit Lertsuwunseri, Jarkarpun Chaipromprasit, Suphot Srimahachota, Wasan Udayachalerm, Srun Kuanprasert, Wacin Buddhari

**Affiliations:** 1 Division of Cardiology, Department of Internal Medicine, Faculty of Medicine, Chiang Mai University, Chiang Mai, Thailand; 2 Division of Cardiology, Department of Medicine, King Chulalongkorn Memorial Hospital, 1873 Rama IV Rd., Pathumwan, Bangkok, 10330 Thailand

**Keywords:** Ultrathin-strut, Sirolimus, All-comer, Registry

## Abstract

**Background:**

Despite numerous studies supporting the outperformance of ultrathin-strut bioresorbable polymer sirolimus-eluting stent (Orsiro SES, Biotronik AG), the generalizability of the study results remains unclear in the Asian population. We sought to evaluate the clinical outcomes of the Orsiro SES in unselected Thai population.

**Methods:**

The Thailand Orsiro registry was a prospective, open-label clinical study evaluating all patients with obstructive coronary artery disease implanted with Orsiro SES. The primary endpoint was target lesion failure (TLF) at 12 months. TLF is defined as a composite of cardiac death, target vessel myocardial infarction (TVMI), emergent coronary artery bypass graft (CABG), and clinically driven target lesion revascularization (CD-TLR). Patients with diabetes, small vessels (≤ 2.75 mm), chronic total occlusions (CTOs), and acute myocardial infarction (AMI) were pre-specified subgroups for statistical analysis.

**Result:**

A total of 150 patients with 235 lesions were included in the analysis. Half of the patients (53.3%) presented with AMI, and 24% had diabetes. Among 235 lesions, 93(39.4%) were small vessels, and 24(10.2%) were chronic total occlusions. The primary endpoint, TLF at 12 months, occurred in eight patients (5.3%), predominately caused by cardiac death. By contrast, the incidences of TVMI and CD-TLR were null. The outcomes in pre-specified subgroup were not different from the overall population (all *p* > 0.05). One definite late stent thrombosis(0.7%) was incidentally observed during primary percutaneous coronary intervention to the non-target vessel.

**Conclusion:**

The safety and efficacy of the ultrathin strut sirolimus-eluting stent in unselected cases are confirmed in the Thailand Orsiro registry. Despite the high proportion of pre-specified high-risk subgroups, the excellent stent performance was consistent with the overall population.

*Trial Registration* TCTR20190325001.

## Background

Drug-eluting stents (DES) are a preferred treatment in patients with obstructive coronary artery disease(CAD) with coronary anatomy suitable for percutaneous coronary intervention (PCI) (1). One of the remarkable DES modification was the reduction of strut thickness with the preservation of radial strength (2). The reduction of strut thickness has mitigated vascular injury and promoted early strut coverage. Multiple meta-analyses have repeatedly reported that ultrathin strut DESs (defined as strut thickness < 70 µm) were associated with a lower incidence of target lesion failure (TLF) in comparison with contemporary second-generation DES (3–6).

The Orsiro bioresorbable polymer sirolimus-eluting stent (Orsiro SES) (Biotronik AG, Bülach, Switzerland) is one of the ultrathin stents platforms. The unique feature of Orsiro SES is a hybrid coating consists of passive and active coating. The passive coating is a layer of amorphous silicon carbide that reduces tissue inflammation and prevents thrombus adhesion (7, 8). The active coating is a layer of bioresorbable drug-polymer that released sirolimus. The clinical benefit of Orsiro SES has been widely demonstrated in many randomized controlled trials (9–13) and national registries (14–16). Recently, Orsiro SES has shown a superior reduction of TLF and late or very late stent thrombosis at the three-year follow-up compared with durable-polymer everolimus-eluting stents (9). In addition, the real-world clinical performance of Orsiro SES also showed favorable medium-(17) and long-term (18, 19) clinical outcomes.

Although numerous studies support the outperformance of the ultrathin strut bioresorbable polymer SES (9), the generalizability of the study results remains unclear in the Asian population since the data was predominantly derived from European centers. It would be of interest to investigate the safety and efficacy of the ultrathin strut BP-SES in real-world Asian population and predefined subgroups: diabetes patients, small vessels (≤ 2.75 mm), chronic total occlusion (CTO), and acute myocardial infarction (AMI).

## Methods

### Study design and population

Thailand Orsiro registry was a prospective, non-randomised, multi-center, observational study to evaluate the safety and clinical performance of the Orsiro SES (Biotronik AG, Bülach, Switzerland) in real-world practice. The study was conducted at two high-volume centers in Thailand: King Chulalongkorn Memorial Hospital, Bangkok and Maharaj Nakorn Chiang Mai Hospital, Chiang Mai. The inclusion criteria were age ≥ 21 years, symptomatic coronary artery disease, signed informed consent, and willingness to participate in all follow-up assessments. The exclusion criteria were patients with known intolerance to antiplatelet/anticoagulant therapy required for PCI, stainless steel, sirolimus or contrast media, planned surgery within six months of PCI unless dual antiplatelet therapy was maintained through the operation, pregnancy and participating in another study before the primary endpoint was assessed. The study protocol was approved by all institutional ethics committees and registered with the Thai Clinical Trials Registry (TCTR), Number TCTR20190325001.

### Device description

The Orsiro SES is an ultra-thin cobalt-chromium stent with hybrid coating. The strut thickness is 60 µm for 2.25 to 3.0 mm and 80 µm for > 3.0 mm. The stent’s outermost is coated by poly-L-lactic acid(PLLA) polymer with an asymmetrical thickness of 7.5 µm on the abluminal stent surface and 3.5 µm on the luminal surface (20). The PLLA carries a sirolimus concentration of 1.4 µm/mm^2^; more than 80% of the drug will elute from the stent over a 90-day. The PLLA slowly degrades via the Krebs cycle causing the polymer breaks down into carbon dioxide and water and completely degrades over 12–15 months (21). After bioresorption is complete, the innermost part of the strut with a passive coating layer of amorphous silicon carbide (PROBIO®) remains and acts as a diffusion barrier to reduce ion release, consequently, reduce the risk of inflammation.

### Procedure and follow-up

The severity of the lesion and lesion length were assessed by visual estimation. PCI was performed using standard interventional techniques. The medication, pre- and post-dilation devices were left to the operator’s discretion. All patients with chronic coronary syndrome (CCS) and acute coronary syndrome (ACS) were required to received dual antiplatelet therapy (DAPT) for a period of 6 and 12 months, respectively. The follow-up was conducted via telephone interview or clinical visit at 6- and 12-month post stent implantation. To ensure data quality, 100% of the data was monitored on-site for endpoint-related data.

### Endpoints

The primary endpoint was target lesion failure (TLF) at 12 months, defined as the composite of cardiac death, target vessel myocardial Infarction (TVMI), clinically driven target lesion revascularization (CD-TLR) and emergent coronary artery bypass graft (CABG).

Secondary endpoints included TLF at six months, target vessel revascularization (TVR), TLR and stent thrombosis according to the academic research consortium (ARC) at 6 and 12 months, clinical device success and clinical procedural success.

Cardiac death is defined as any death due to proximate cardiac cause (e.g. MI, low-output failure, fatal arrhythmia), unwitnessed death or death of unknown cause and all procedure-related deaths, including those related to concomitant treatment. Target vessel myocardial infarction defined as target vessel Q-wave or non-Q-wave myocardial infarction. Myocardial infarction was adjudicated according to the universal definition of myocardial infarction (22). Periprocedural myocardial infarction is defined as an increased in cardiac troponin greater than 3 × 99th percentile URL in subjects with normal baseline troponin values. However, the protocol did not mandate to routinely collect the biomarkers pre- and post-procedure. Clinically driven target lesion revascularization is defined as any repeat revascularization of the target lesion with demonstration of a positive functional clinical investigation, ischemic electrocardiography changes at rest in a distribution consistent with the target vessel, or ischemic symptoms and in-lesion diameter stenosis ≥ 50% by quantitative coronary angiogram. Target vessel revascularization is defined as any repeat percutaneous intervention or surgical bypass of any segment of the target vessel including the target lesion.

Clinical device success was defined as post-procedural residual stenosis of less than 20% by visual estimation and without the use of a device outside the assigned treatment strategy. Clinical procedural success was defined as device success without ischemia-driven major adverse cardiac event (MACE) during the hospital stay up to the first seven days post-index procedure. Major adverse cardiac event (MACE) is defined as the composite of cardiac death, TVMI, CD-TLR, and emergent CABG.

In the case of multiple lesions treatment, all treated lesions must meet the procedural success definition. Study data were collected and managed using REDCap electronic data capture tools hosted at Chiang Mai University.

### Statistical methods

Four subgroups were pre-defined for statistical analysis; i) diabetes patients, small vessels (≤ 2.75 mm), chronic total occlusion (CTO), and acute myocardial infarction (AMI).

Categorical variables were reported with frequencies and percentages. The continuous variables were summarized as mean ± standard deviation (SD) or median and interquartile ranges (IQR 1st-3rd) as appropriate. The primary and secondary endpoints were analyzed based on the intention-to-treat population. Kaplan–Meier method was used to demonstrate the time to event of the primary endpoint. The comparison among the predefined subgroups was performed with the log-rank test. A two-sided *p*-value < 0.05 was considered statistically significant. Statistical analysis was performed by IBM SPSS Statistics version 26.0 (IBM Corp, Armonk, NY, USA).

## Results

### Patients characteristics

**Fig. 1:**
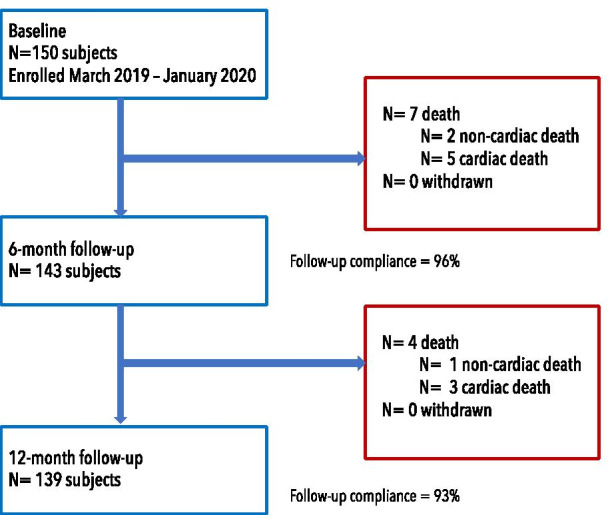
Study flow chart

A total of 150 patients were included between March 2019 and January 2020 (Fig. 1). The baseline patient characteristics of the entire cohort and prespecified subgroups were summarized in Table 1. The mean age of the patients was 67 ± 12 years, 68% were male, and 32% had diabetes. The proportion of patients with ACS (53.3%) was numerically higher than patients with CCS (46.7%). On average, 18% of the patients had chronic kidney disease (defined as estimated glomerular filtration rate < 60 ml/min/1.73 m^2^) and previous myocardial infarction. Among patients presenting with ACS, cardiogenic shock was noted in 2 patients (1.3%). Of 80 culprit lesions, 19 lesions (23.8%) demonstrated thrombus. DAPT was continued until 12 months in 92.0% of the patients.

**Table 1:** Baseline clinical characteristics

	All population	Diabetes	Small vessel	CTO	AMI
	N = 150	N = 48	N = 75	N = 17	N = 80
Age, years	67.2 ± 12.5	69.7 ± 10.0	65.8 ± 13.6	65.4 ± 15.3	65.2 ± 13.7
Men	102(68.0)	30(63.8)	46(62.2)	11(64.7)	57(71.3)
Current smokers	20(13.3)	3(6.4)	10(13.5)	4(23.5)	17(18.8)
Diabetes	48(32.0)	n/a	27(36.5)	6(35.3)	21(26.3)
Hypertension	113(75.3)	42(89.4)	58(78.4)	13(76.5)	56(70.0)
Hyperlipidemia	96(64.0)	34(72.3)	49(66.2)	10(58.8)	42(52.5)
Chronic kidney disease	27(18.0)	15(31.9)	12(16.2)	5(29.4)	15(18.8)
Chronic obstructive pulmonary disease	5(3.3)	1(2.1)	1(1.4)	1(5.9)	2(2.5)
Previous coronary revascularization	27(18.0)	12(25.5)	16(21.6)	5(29.4)	6(7.5)
Previous myocardial infarction	27(18.0)	11(23.4)	12(16.2)	3(17.6)	13(16.3)
History of heart failure	18(12.0)	6(12.8)	10(13.5)	3(17.6)	10(12.5)
History of cancer	3(2.0)	1(2.1)	3(4.1)	2(11.8)	2(2.5)
Chronic coronary syndrome	70(46.7)	26(55.3)	40(54.1)	8(47.1)	0(0.0)
Acute coronary syndrome	80(53.3)	21(44.7)	34(45.9)	9(52.9)	80(100.0)
ST segment elevation MI	40(50.0)	6(28.6)	19(55.9)	6(66.7)	40(50.0)
Non ST segment elevation MI	40(50.0)	15(71.4)	15(44.1)	3(33.3)	40(50.0)
Medication at discharge					
Aspirin	147(98)	45(95.7)	72(97.3)	16(94.1)	78(97.5)
Clopidogrel	89(59.7)	33(70.2)	44(59.5)	9(52.9)	36(45.6)
Ticagrelor	49(33.8)	13(28.3)	23(32.9)	4(23.5)	38(48.7)
Prasugrel	11(7.5)	1(2.2)	7(9.9)	3(17.6)	5(6.4)
Statin	140(94.0)	44(93.6)	69(93.2)	16(94.1)	72(91.1)
DAPT at 12 months	121(87.1)	36(83.7)	60(88.2)	15(100.0)	69(92.0)

Data shown in n(%) or mean ± SD

*AMI* acute myocardial infarction; *CTO* chronic total occlusion; *DAPT* dual antiplatelet therapy

### Angiographic and procedural characteristics

Among 150 patients, 212 lesions were treated with study stents at the index procedure. Seventeen patients with 23 lesions were further scheduled for stage PCI, with the median interval from index PCI to the stage PCI date was 38.0 (IQR 27.5–66.3) days. Finally, 235 lesions were treated with Orsiro SES stents. Baseline angiographic characteristics were tabulated in Table 2. The left anterior descending artery (51.1%) was frequently treated with the study stent followed by the right coronary artery (26.0%), left circumflex (18.7%) and left main (4.3%), respectively. Nearly three-quarters of the lesions were type B2/C (72.5%), and 20.9% were bifurcation lesions. Of all treated lesion, small vessels and CTO were observed in 39.4% and 10.2%, respectively.

**Table 2:** Angiographic characteristics

	All population
	n = 150, L = 235
Vascular access	
Femoral	129/169
Radial	38/169
Brachial	2/169
Target vessel	
Left anterior descending	120(51.1)
Left circumflex artery	44(18.7)
Right coronary artery	61(26.0)
Left main	10(4.3)
AHA/ACC lesion classification	
A/B1	65(27.5)
B2/C	171(72.5)
Preprocedural TIMI 3	164(69.5)
No. of target lesions (per patient)	
1	92(61.3)
2	36(24.0)
3	15(10.0)
4	5(3.3)
5	2(1.3)
Moderate to heavy calcification*	38(16.1)
Bifurcation lesion	49(20.9)
Moderate to excessive tortuous vessel	30(12.7)
Eccentric lesion	187(79.2)
Lesion with thrombus	13(5.5)
Chronic total occlusion	24(10.2)
Obstruction length, mm	22.2 ± 10.8
Reference lumen diameter, mm	2.93 ± 0.45
Small vessel ≤ 2.75 mm	93(39.4)

Data shown in n(%) or mean ± SD *Moderate calcification is defined as radiopacities noted only during the cardiac cycle before contrast injection. Severe calcification is defined as radiopacities seen without cardiac motion before contrast injection. *TIMI* Thrombolysis in Myocardial Infarction

Table 3 summarizes the procedural characteristics. Predilation was performed in 91.9% of the treated lesion and predominately performed with semi-compliance balloon 72.5%. Study stents were implanted in the lesion with a mean reference vessel diameter of 2.93 ± 0.45 mm and length of 22.2 ± 10.8 mm. Post-dilation was conducted in 69.9% and usually done with non-compliance balloon with the mean inflation pressure of 16.5 ± 4.0 atmosphere and maintain pressure at 23.0(IQR 13.3–38.8) seconds. Among 49 bifurcation lesions, 47 lesions (95.9%) and 2 lesions (4.1%) were treated with provisional and two-stent technique, respectively. Intracoronary imaging-guided PCI was employed in 6.8% of the lesions. Clinical device success was 100% (235/235) of the lesions with clinical procedural success was 98% (147/150) of the patients. Three patients failed to achieve clinical procedural success due to the cardiac death on days 1,4,5 after index procedure. All of them presented with acute ST-elevation myocardial infarction (STEMI).

**Table 3:** Procedural characteristics

	All population
	n = 150, L = 235
Diameter stenosis, %	84.9 ± 11.8
Pre-dilation	
Pre-dilatation	217(91.9)
Cutting or scoring balloon	10(4.2)
Non-compliance balloon	35(14.8)
Semicompliance balloon	171(72.5)
Nominal pre-dilation balloon diameter, mm	2.49 ± 1.00
Pre-dilation balloon length, mm	17.1 ± 4.9
Stent procedure	
No. of study stents implanted	
Per patient	1.7 ± 1.1
Per lesion	1.1 ± 0.3
Per vessel	1.4 ± 0.7
Total length of implanted stents, mm	26.9 ± 12.2
Mean nominal device diameter, mm	2.97 ± 0.46
Overlapping stents	18(7.6)
Bifurcation lesions treated with provisional stenting	47(95.9)
Bifurcation lesions treated with two-stent technique	2(4.1)
Number of stents per lesion	
1	217(92.3)
2	16(6.8)
3	1(0.4)
4	1(0.4)
Intracoronary imaging in the procedure	16(6.8)
Post-dilation	
Post-dilation	165(69.9)
Nominal post-dilation balloon diameter, mm	3.12 ± 0.53
Post-dilation balloon length, mm	15.5 ± 6.2
NC used for post-dilation	110(67.1)
Max applied pressure, atm	16.5 ± 4.0
Cumulative inflation time	23.0(13.3–38.8)
Device success	235(100)
Procedure success	147(98.0)

Data shown in n(%) or mean ± SD or median (interquartile range 1st–3rd)

### Clinical outcomes

**Fig. 2:**
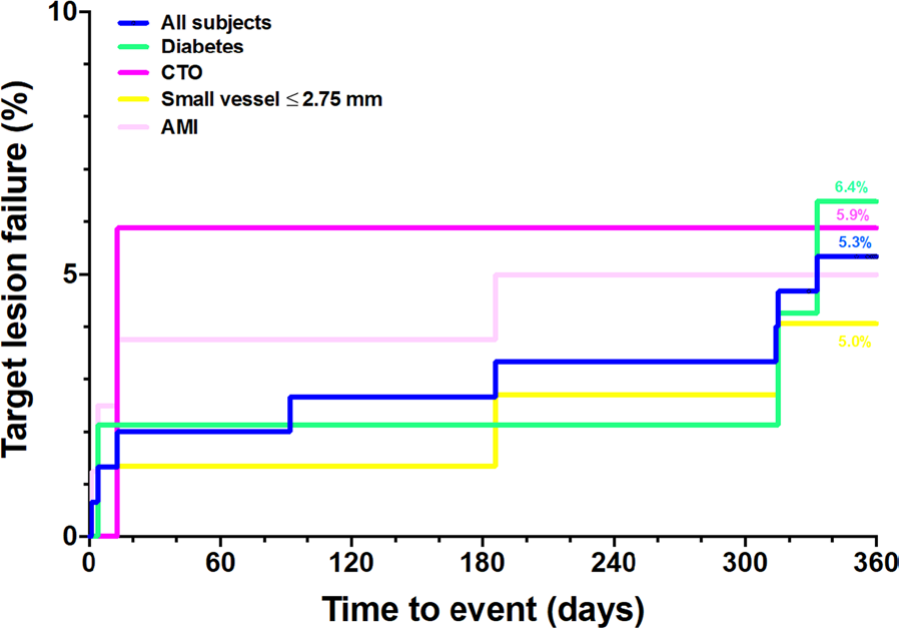
Kaplan–Meier event estimates for target lesion failure

Clinical data at 6 months and 12 months was available in 96% and 93% of the patients, respectively (median follow-up duration was 372 days [IQR 367–380]). The primary endpoint, TLF at 12 months, occurred in eight patients (5.3%), predominately caused by cardiac death. Among eight patients who died, half of them presented with ST-elevation myocardial infarction and died after the PCI procedure on days 1 to 13 (three cases died from the complications related to AMI and one case from acute decompensated heart failure). In pre-specified subgroups, the TLF at 12 months was 6.4% in diabetic patients, 4.1% in small vessels, 5.9% in CTOs and 5.0% in AMI patients (Fig. 2). All clinical outcomes were detailed in Table 4. Interestingly, the occurrence of TVMI and CD-TLR were null. One patient received TVR at day 265, two patients had definite or probable stent thrombosis. One probable subacute stent thrombosis was suspected due to unexplained death at home after being discharged from hospital one-day post-procedure. One definite late stent thrombosis was incidentally observed during coronary angiography for primary PCI to the non-target vessel at day 333.

**Table 4:** Adverse cardiac events at 6 and 12 months

Event	Total events at 6 months	Total events at 12 months	Diabetes	Small vessel	CTO	AMI
	n = 150	n = 150	N = 48	N = 75	N = 17	N = 80
Target lesion failure	5(3.3)	8(5.3)	3(6.4)	3(4.1)	1(5.9)	4(5.0)
Cardiac death	5(3.3)	8(5.3)	3(6.4)	3(4.1)	1(5.9)	4(5.0)
Target vessel myocardial infarction	0(0.0)	0(0.0)	0(0.0)	0(0.0)	0(0.0)	0(0.0)
Emergent CABG surgery	0(0.0)	0(0.0)	0(0.0)	0(0.0)	0(0.0)	0(0.0)
Clinically driven target lesion revascularization	0(0.0)	0(0.0)	0(0.0)	0(0.0)	0(0.0)	0(0.0)
All-cause death	7(4.7)	11(7.3)	1(2.1)	6(8.1)	2(11.8)	5(6.3)
All myocardial infarction	1(0.7)	2(1.3)	0(0.0)	0(0.0)	0(0.0)	0(0.0)
Non-target vessel myocardial infarction	0(0.0)	1(0.7)	0(0.0)	0(0.0)	0(0.0)	0(0.0)
Target vessel revascularization	0(0.0)	1(0.7)	0(0.0)	0(0.0)	0(0.0)	1(1.3)
Non-target vessel revascularization	0(0.0)	2(1.3)	1(2.1)	0(0.0)	0(0.0)	1(1.3)
Definite or probable stent thrombosis	1(0.7)	2(1.3)	1(2.1)	0(0.0)	0(0.0)	1(1.3)
Definite	0(0.0)	1(0.7)	1(2.1)	0(0.0)	0(0.0)	0(0.0)
Probable	1(0.7)	1(0.7)	0(0.0)	0(0.0)	0(0.0)	1(1.3)

Data shown in n(%). Abbreviation as Table 1; *CABG* coronary artery bypass graft

## Discussion

The present study investigated the Thai experience with ultrathin BP-SES in real-world practice. The main findings of the study were as follows: 1) Orsiro SES implantation in Thai patients has TLF rate of 5.3% that was consistent with previous reports; 2) the outcomes in the prespecified subgroup were not different from the control population.

The Orsiro SES platform has been studied in > 50,000 patients. The clinical outcomes of the Thailand Orsiro registry were concordant with several studies both in registries and RCTs. The TLF rates of the present study were 5.3% compared to 5.1% in all-comer registry BIOFLOW-III (17) (Real-world experience with a novel biodegradable polymer sirolimus-eluting stent), and 6% in BIOFLOW-V (10) (Ultrathin, bioresorbable polymer sirolimus-eluting stents versus thin, durable polymer everolimus-eluting stents in patients undergoing coronary revascularization) randomized trial. Thus, the TLF rates of the present study align with previous data.

However, other RCTs reported the TLF rates less than 5%; the incidence ranged from 3.2% to 4.2% (11, 12, 23–25). The population included in the RCTs were selected population and, by design has excluded poor prognosis patients; these might explain the low TLF rates in RCTs compared to the present study.

Notably, the primary outcomes of the present study were driven by cardiac death in which the majority of them occurred as the complications of the initial presentation with AMI. Post-discharge cardiac death within 14 days were 3 patients (2.0%). By contrast, the major events in other trials were TVMI or TLR with the low incidences of cardiac death of 0% to 1.3% (10, 11, 17, 23). The high incidence of cardiac death in the present study might be explained by the half of the population was ACS patients. Though the previous publications have reported the proportion of ACS patients from 32.5% to 53%, it should be noted that the patients with MI in such trials were eligible for enrollment after MI onset > 72 h (10, 11). The inclusion criteria were in contrast to the present study that enrolled all patients who received Orsiro SES without the limitation of MI onset. To date, two trials were testing the safety and efficacy of Orsiro in STEMI patients; HEROES (16) (Hard Events AfteR Orsiro Sirolimus-Eluting Stent in STEMI: A Multicenter Registry) and BIOSTEMI (12) (Biodegradable polymer sirolimus-eluting stents versus durable polymer everolimus-eluting stents in patients with ST-segment elevation myocardial infarction) randomized trial, the cardiac death rates at 12 months were 4.8% and 3%, respectively. This all-comer study has reproducible results as in the dedicated ACS trial.

The absence of TVMI and CD-TLR events in the current study is deserved for discussion. The BIOFLOW-V (10) trial has reported TVMI rates of 5%, in contrast to the present study that was null. The difference might be explained by the study protocol that did not intended to capture periprocedural MI events. Besides, periprocedural MI in the ACS setting, especially STEMI, was difficult to determine in real-world practice. The zero events of CD-TLR were lower than that reported 3.0% in all-comer registry BIOFLOW-III (17), 2.0% in randomized controlled trials (10, 11, 23, 26) except 1.0% in BIOSTEMI (12) trial. Low incidence of TLR might be explained by meticulous post-dilation performed in the present registry. According to the BIOFLOW-III (17) registry, post-dilation was performed only 25.3% whereas the post-dilation rates of the Thailand Orsiro registry were 69.9% with a high proportional of non-compliance balloon (67.1%) at high pressure (16.5 atmosphere). In addition, the registry protocol did not determine to perform follow-up angiography; therefore, the incidence of TLR might be underreporting. However, in real-world practice, repeat revascularization will be performed only in patients who present with ischemic symptom. The current analysis provides real-world evidence of TLR rates in unselected patients.

The incidence of stent thrombosis (definite and probable) of the present study was 1.3% and higher than previous reports (< 1%) (10, 11, 17, 23, 26) except in the BIOSTEMI (12) trial that was 2%. A higher rate of stent thrombosis in the present study may be explained from 1) a small sample size compared to the previous publications (10, 11, 17, 23, 26); 2) the definition of stent thrombosis followed the first Academic Research Consortium (ARC). The probable stent thrombosis was counted in one case due to unexplained death within the first 30 days (27). If the update ARC-2 criteria (28) were applied in the current study, no case would have probable stent thrombosis since the criteria utilized any myocardial infarction that was related to the territory of the implanted stent without angiographic confirmation of stent thrombosis, regardless of the time after the index procedure (28). However, the incidence of definite stent thrombosis (0.7%) was comparable to other trials and still unchanged regardless of the version of ARC definition.

Regarding the regional and ethnic differences, the primary endpoint of the Thailand Orsiro registry is in line with the TLF rate of 4.6% from the BIOFLOW-III Italian Satellite Registry (14). On the contrary, the incidence of TLF was 2.8% in the BIOFLOW-III Canada registry (15), 2.2% in the Japanese subgroup analysis from the BIOFLOW-IV (11) randomized trial. The disparity of Thai patients’ TLF rates to other ethnic groups might be explained by the higher proportion of complex lesion (type B2/C) and the presentation of acute coronary syndrome.

### Limitations

The present study has several limitations to be addressed. First, the pre- and post-procedural quantitative coronary angiography was not mandated in the protocol. Consequently, the baseline lesion characteristics were based on operator discretion. Second, there was no protocol mandated for pre-dilation, post-dilation according to the study objective. The operator experience would undoubtedly affect the immediate PCI outcomes. However, the study results reflected the real-world practice outcomes when Orsiro SES were implanted in unselected patients and operators. Third, the sample size was small in comparison to previous national registries of the similar platform. In addition, the present study did not have the power to detect differences of the pre-specified subgroup; thus, the results must be interpreted with caution. Fourth, comparison of the number of events to previous publications should be cautiously interpreted due to varied patient clinical settings, different endpoints, and definitions in an individual trials.

## Conclusions

The safety and efficacy of the ultrathin strut sirolimus-eluting stent in unselected patients are confirmed in the Thailand Orsiro registry. Despite the high proportion of pre-specified high-risk subgroups, the excellent stent performance was consistent with the overall population.

## Data Availability

The datasets used and/or analyzed during the current study are available from the corresponding author on reasonable request.

## Abbreviations

ACS: Acute coronary syndrome
BP-SES: Bioresorbable polymer sirolimus-eluting stent
CABG: Coronary artery bypass graft
CAD: Coronary artery disease
CD-TLR: Clinically driven-target lesion revascularization
CTO: Chronic total occlusion
DES: Drug-eluting stent
PCI: Percutaneous coronary intervention
SES: Sirolimus eluting stent
STEMI: ST-elevation myocardial infarction
TLF: Target lesion failure
TVMI: Target vessel myocardial infarction
